# A Comprehensive Review of Emulsion-Based Nisin Delivery Systems for Food Safety

**DOI:** 10.3390/foods14081338

**Published:** 2025-04-13

**Authors:** John Kapolos, Dimitra Giannopoulou, Konstantinos Papadimitriou, Athanasia Koliadima

**Affiliations:** 1 Department of Food Science and Technology, School of Agriculture and Food, University of the Peloponnese, 24100 Kalamata, Greece; i.kapolos@uop.gr; 2 Department of Chemistry, University of Patras, 26504 Patras, Greece; dgianno_9@yahoo.gr; 3 Laboratory of Food Quality Control and Hygiene, Department of Food Science and Human Nutrition, Agricultural University of Athens, Iera Odos 75, 11855 Athens, Greece; kpapadimitriou@aua.gr

**Keywords:** food safety, bacteriocin, nisin, delivery system, emulsion, nanoemulsion, microemulsion, encapsulation, physicochemical characterization

## Abstract

Foodborne diseases are one of the most serious problems the food sector has to confront, while questions have been raised concerning the effects of several antimicrobial additives on consumer health. Nisin is a peptide produced primarily by *Lactococcus lactis* with antimicrobial properties mostly against Gram-positive bacteria. It is generally recognized as safe (GRAS) for use in a wide range of food categories. However, its interaction with components of the food matrix, its susceptibility to proteolytic degradation, or the competitive presence of other components may limit its activity. To enhance its effectiveness against Gram-negative bacteria, its combination with essential oils or other antimicrobial components has been investigated. In addition, its encapsulation in several types of nano-delivery systems has been used to protect nisin from food matrix sequestering while regulating its release. In this review, we present how nisin is utilized, alone or in combination with other antimicrobial agents in a range of emulsion types, as well as the standard techniques for the physicochemical characterization of these systems.

## 1. Introduction

Food production, storage, and distribution have changed drastically in recent decades. These are dictated by technological advancements, and the globalization of the food industry [1]. In parallel, consumer preferences have shifted at least partly towards fresh, unprocessed food that is safe and beneficial to health. Frozen or packed food, ready-to-eat meals, pre-cut salads, fast food, and other products satisfy the growing demand for variety and simplicity of meal preparation [2]. However, foodborne diseases remain a major challenge for public health and, in several instances, may lead to fatalities [3]. In addition, foodborne diseases result in significant economic burdens due to hospitalization, product recalls, legal liabilities, etc. [4].

The term biopreservation or biocontrol is defined as the use of microorganisms or their products that exhibit antimicrobial properties against food spoilage microorganisms and foodborne pathogens. It is an effective approach to extend shelf life and improve food safety [5,6]. Several antimicrobial food additives must be used with caution due to concerns about their influence on consumer health. A variety of preservatives, including nitrates, sulfites, parabens, BHA (Butylated hydroxyanisole), BHT (Butylated hydroxytoluene), and others, have been associated with adverse effects such as hypersensitivity, allergy, asthma, hyperactivity, neurological damage, and cancer [7].

As a result, the food industry has focused on research for natural antimicrobial compounds including antimicrobial peptides (AMPs), essential oils (EOs), phenolic compounds, organic acids, biopolymers such as chitosan, etc. [8]. Bacteriocins are members of AMPs produced by both Gram-negative and Gram-positive bacteria [5]. Bacteriocins are ribosomally synthesized with a relatively narrow antimicrobial spectrum, being active mostly against species/strains phylogenetically close to the producer strains [5,9]. Given the perceived safety status of many lactic acid bacteria (LAB) species according to different regulatory organizations (e.g., Food and Agriculture Organization (FAO)/World Health Organization (WHO) and the European Food Safety Authority (EFSA)) due to their long use in food fermentations or as commensals, extensive research has focused on the bacteriocins they produce [10,11]. Nisin is the best-studied LAB bacteriocin and it has widespread commercial applications. This review focuses on the development of emulsions for the delivery of nisin in foods to circumvent the neutralization or sequestration of this AMP that may occur during its direct addition to the food matrix.

## 2. Nisin—Basic Facts and Physicochemical Properties

**Figure 1:**
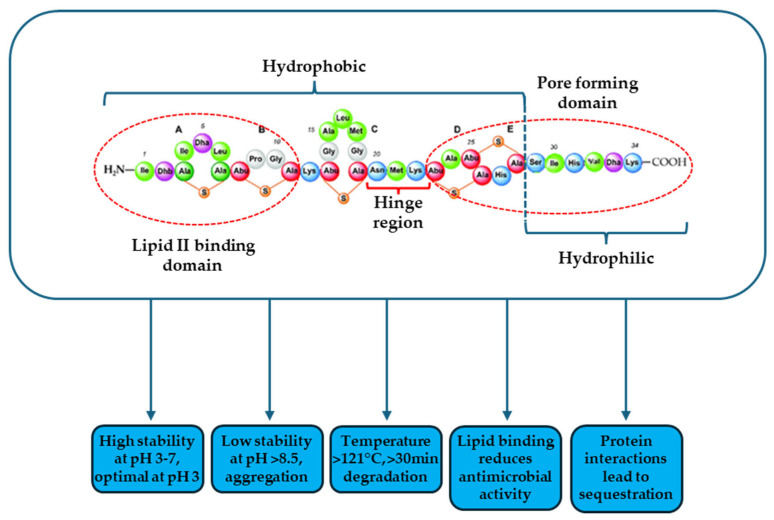
Schematic representation of nisin A molecule integrating important physicochemical and functional properties.

Nisin was first discovered in 1928 as an antimicrobial peptide (AMP) produced by *Streptococcus lactis* (now known as *Lactococcus lactis*). Its antimicrobial activity was initially observed against *Lactobacillus bulgaricus* cells [12]. Nisin is an amphiphilic peptide composed of 34 amino acids, with a molecular mass of 3354 Da (Figure 1). It is ribosomally synthesized and contains unique post-translational modifications, including dehydroalanine (Dha) and dehydrobutyrine (Dhb), which are derived from the dehydration of serine and threonine, respectively. Additionally, nisin features the rare amino acids lanthionine and β-methyllanthionine, which form thioether bridges, contributing to its distinctive structure [13,14,15]. Thioether bridges form five ring structures (identified as rings A, B, C, D, and E). As a result, nisin has two rigid ring systems (A, B, C rings and D, E rings) located at the amino-terminal and carboxy-terminal ends of the molecule, respectively. A hinge region (from the 20th to the 22nd amino acid)—frequently found in antimicrobial peptides—divides the two ring systems, giving the molecule flexibility. These rings support the three-dimensional structure of the nisin molecule and contribute to its amphiphilic character [13]. Because of its structure, nisin appears to be positively charged overall and exhibits thermostability as well as bactericidal activity.

Nisin was initially found in two physical forms: nisin A and nisin Z. Over time, additional variants of nisin have been mentioned, including nisin F, Q, U, U2, and P, exhibiting compositional and structural differences compared to nisin A and Z [16,17,18,19]. Today, several natural variants of nisin have been discovered, and many more have been synthesized in the laboratory. Nisin A and Z vary by the amino acid at position 27, where a histidine in nisin A is substituted by asparagine in nisin Z. The difference appears not to affect thermal stability, tolerance to pH changes, susceptibility to proteolytic enzymes, and the overall spectrum of antibacterial activity. However, at neutral pH, nisin Z is more soluble than nisin A due to asparagine’s stronger polar side chain than histidine. This structural alteration does not affect antibacterial activity, but it does change physicochemical properties: nisin Z is more soluble and has better dispersion when used in foods [20].

The effect of pH on nisin solubility is significant. It has an isoelectric point above 8.5 and, therefore is cationic at neutral pH [21]. Liu and Hansen examined the solubility of nisin at pH 2 to 12. The solubility fell sharply and consistently from 57 mg/mL at pH 2 to 1.5 mg/mL at pH 6, then to 0.25 mg/mL at pH 8.5, before leveling out. Under alkaline conditions nucleophiles present can modify Dha and Dhb, leading to lower solubility and instability and the formation of multimers [13,22,23]. Similarly, its antibacterial activity is higher at acidic pH (for example, hydrochloric acid solution—pH 2.5), and declines with increasing pH values [22].

Nisin remains stable at low temperatures (for example, during freezing), but it can become inactive when exposed in heat for an extended period of time. Furthermore, nisin thermostability is closely correlated with pH. For example, nisin retains antibacterial activity better at acidic pH and is most stable at pH 3, where it withstands autoclaving at 121 °C. In contrast, it loses activity after 30 min at 63 °C, at pH 11 [24]. Davies et al. also discovered that a nisin solution (10,000 IU mL^−1^) lost just 5% of its activity after 20 min at 115 °C and 15% after 15 min at 121 °C and was most stable to autoclaving at pH 3. Solutions with pH values farthest from pH 3 show a considerable drop in activity [25].

Nisin’s primary antibacterial activity is against Gram-positive bacteria. It has been reported to be effective against important Gram-positive foodborne pathogens, like *Listeria monocytogenes* and *Staphylococcus aureus*, the spores of *Clostridium* and *Bacillus*, as well as the thermophilic species of *Thermoanaerobacterium* and *Geobacillus* [13,15,26,27]. Research has shown that nisin binds to the bacterial plasma membrane and kills cells by causing the formation of pores. The main mechanism relies on the binding of the hydrophobic part of nisin to lipid II. Lipid II participates in the synthesis of peptidoglycans of the bacterial cell wall. Next, the C-terminal end, with the help of the hinge part, penetrates the membrane to form a pore with other nisin molecules [28]. The formation of pores causes the efflux of intracellular molecules such as ions, amino acids or ATP, resulting in the immediate disruption of all cellular biosynthetic processes. The entry of nisin into the bacterial cell also inhibits the synthesis of various macromolecules such as DNA, RNA, proteins and polysaccharides, as well as the deterioration of the electrochemical potential of the membrane [29,30]. Nisin is typically not active against Gram-negative bacteria mostly due to their outer membrane that does not allow for access to lipid II.

Nisin’s safety profile has been extensively studied, focusing on toxicity, cytotoxicity against human cells, and potential long-term adverse effects. A 90-day oral toxicity study in F344 rats evaluated the effects of dietary nisin A. The no-observed-adverse-effect level (NOAEL) was determined to be 5.0% in the diet, equivalent to approximately 3000 mg/kg body weight per day, as no toxicologically significant changes in clinical signs, body weight, food consumption, or organ pathology were observed [31]. The study of O’ Reilly et al. [32] examined the effect of oral administration of nisin on the microflora and metabolic functions of the pig intestine. Nisin survived in the gastrointestinal tract and caused a decrease in Gram-positive bacteria and increase in Gram-negative bacteria, also affecting short-chain fatty acid production (decrease in acetic and butyric and increase in propionic acid). The changes were reversed 10 days after the discontinuation of treatment, showing that nisin can temporarily modulate the microbiome without permanent disturbances or disturbing the growth performance of the animals. On the other hand, long-term administration of a diet containing Nisaplin^®^ to mice resulted in a significant increase in the number of macrophages/monocytes isolated from peripheral blood [33], but this was not directly related to nisin as Nisaplin also contains milk proteins and salt. Research indicates that nisin can induce cytotoxic effects in certain human cell lines in vitro. For instance, nisin exhibited cytotoxic effects on Vero cells (monkey kidney epithelial cells) with an IC50 value of 105 μM. However, it is important to note that these cytotoxic concentrations are significantly higher than those used for antimicrobial purposes in food preservation. The European Food Safety Authority (EFSA) reviewed new toxicological data and proposed an acceptable daily intake (ADI) of 1 mg/kg body weight per day for nisin. This assessment was based on an NOAEL of 225 mg/kg body weight per day [34]. A recent evaluation reaffirmed this ADI, emphasizing that systemic exposure to nisin is unlikely due to its degradation in the gastrointestinal tract [35]. The study of Abd-Elhamed et al. evaluated the toxicity and antifungal activity of nisin in the form of nanoparticles (Nisin NPs) against *Aspergillus flavus* in Ras cheese [36]. Nisin NPs were prepared by nanoprecipitation using acetic acid and characterized by FTIR, TEM and zeta-dynamics. The nanoparticles exhibited an 8-fold lower MIC than pure nisin (0.0313 mg/mL vs. 0.25 mg/mL) and completely inhibited *A. flavus* at week 7 (vs. 10 for nisin). Cytotoxicity to Vero cells was negligible, while the organoleptic characteristics of the cheese remained unaffected, confirming the safety and efficacy of Nisin NPs.

## 3. Factors Limiting Nisin Effectiveness

Nisin in (semi-)purified form has been recognized as a food preservative in over 60 countries and has been licensed by EFSA as E 234 [15,27,34]. It has found commercial and research applications in almost all food categories, including dairy, meat, fish and plant-based products, as well as ready-to-eat and convenience foods. However, the effectiveness of nisin in antimicrobial protection of foods may be reduced by factors such as its binding to food components such as lipids, its uneven distribution between polar and non-polar parts of the food, pH, and conditions that destabilize its biological activity such as proteolytic degradation or oxidation, as well as possible competitive interactions between different preservatives or food ingredients [15,37,38,39].

Bell and Lacy estimated the percentage of unbound and hence active nisin in minced meat and meat emulsion treated with nisin solution. While parameters such as the presence of NaCl and/or NaNO_2_, the degree of meat partitioning, the concentration of nisin/g meat, and the ratio of meat to extraction liquid (HCl, 0.02N) had little or no impact on nisin recovery rate, increasing the quantity of fat in the meat, these increased the percentage of recovered nisin, which was attributed to fat blocking its absorption sites on the meat surface. The addition of nisin at concentrations not exceeding the maximum amount that the meat surface was capable of adsorbing gave a stable recovery rate of about 40%. At higher amounts of added nisin (between 100 and 200 IU/g), the recovery rate was reduced, perhaps due to excess nisin molecules polymerization with those already adsorbed to the meat surface [40].

Henning and colleagues confirmed that typical membrane components such as phosphatidylglycerol, phosphatidylethanolamine, phosphatidylcholine, cardiolipin and the minor component undecaprenyl phosphate were able to antagonize the inhibitory effect of nisin on peptidoglycan synthesis [41]. Another study showed that initial nisin activity (50 U/mL) decreased by more than 88% when added to milk containing 12.9% fat against *L. monocytogenes,* while Tween 80 substantially improved nisin action against *L. monocytogenes* in milk, regardless of the fat content [42]. A similar conclusion was reached by Bhatti et al., who also found that Tween 80, partially compensated for the reduction in nisin activity when added to half-and-half (12.9% milk fat) milk [43].

Phospholipids in milk fat can bind a substantial portion of added nisin, especially after milk homogenization. Milk homogenization causes a fourfold to sixfold increase in the fat/plasma interface, changing the distribution of milk proteins and nisin adsorption to fat globule surfaces, which may interfere with its antimicrobial activity [43,44].

Castro et al. prepared oil-in-water emulsions with a composition similar to salad dressings to evaluate the effect of nisin, potassium sorbate, Tween 20, and oil content on the growth of the *Lactobacillus fructivorans* population. The growth bacteria were inhibited when nisin was added, but its activity was dependent on the system composition. Potassium sorbate did not show bactericidal activity when used alone and at a low oil content (110 g/kg) acted antagonistically to nisin, while at a higher one (230 g/kg or 460 g/kg), it acted synergistically. The addition of Tween 20 to the lower oil content restored nisin activity, while that to the intermediate oil content exerted an antagonistic effect, and that to the higher oil level had no impact on nisin activity [45].

Evidently, nisin’s effectiveness is often reduced due to its interaction with food ingredients, and it may also lose its activity shortly after being applied directly to the food matrix. Various encapsulation techniques can effectively protect nisin from proteolytic degradation and interactions with food matrix components while simultaneously ensuring a controlled release rate. Such encapsulating systems are frequently based on different types of emulsions, which are presented below.

## 4. Emulsions—Terminology, Methods of Preparation and Physicochemical Characterization

Emulsions consist of two phases, oil and water (with low solubility in each other), with one of them forming small droplets dispersed into the other [46]. Therefore, there can be oil-in-water (O/W) or water-in-oil (W/O) emulsions. Multiple emulsions can also be prepared such as water in oil in water (W/O/W) and oil in water in oil (O/W/O) [47]. Based on the radius of the dispersed phase particles emulsions are classified as follows: (macro)emulsions or coarse emulsions with particles radii larger than 100 nm, nanoemulsions with particles less than 100 nm, and microemulsions with particles smaller than 50 nm (typically below 25 nm) [48] (Table 1). In the literature, various upper particle size limits for nanoemulsions have been proposed, such as 500 nm, 200 nm, 30 nm, and 100 nm. However, these figures are frequently presented without indicating whether they pertain to radius or diameter [49]. There are also the Pickering emulsions that are stabilized by solid particles instead of surfactants [50].

**Table 1:** Comparison of size, stability, and physicochemical properties of the different types of emulsions according to [46,47,48,49,50].

Property	Macroemulsions	Nanoemulsions	Microemulsions	Pickering Emulsions
Size (Radius)	100 nm–100 µm	10–100 nm	2–100 nm	0.1–100 µm
Shape	Spherical	Spherical	Spherical, lamellar rod micelles or sponge-like	Spherical
Stability	Thermodynamically unstable, weakly kinetically stable	Thermodynamically unstable, kinetically stable	Thermodynamically stable	Highly stable due to irreversible adsorption of particles
Polydispersity Index	Often high (0.2–0.5)	Typically low (0.1–0.3)	Typically low (0.1–0.3)	Typically low (0.1–0.3)
Stabilization Mechanism	Surfactants	Surfactants	Surfactants and co-surfactants	Solid particles (e.g., silica, proteins, cellulose, bacteria)
Transparency	Opaque	Translucent/transparent	Transparent	Opaque

Macroemulsions or coarse emulsions are thermodynamically unstable but can be kinetically stabilized using surfactants, polymers, or solid particles, such as in Pickering emulsions. Macroemulsions require energy input, such as stirring. Temperature, pH, and ionic strength all have an impact on their stability, which is controlled by repulsive forces that prevent droplet coalescence over time [51,52,53].

Nanoemulsions (also known as submicron or mini emulsions) are thermodynamically unstable systems composed of an oily phase, water, and a surfactant. Nanoemulsions scatter visible light slightly; so, they appear clear or slightly opaque. They require a relatively small amount of surfactant 3–8% *w*/*w*. Nanoemulsions provide potential benefits for specific applications due to their tiny particle size, including improved long-term stability, great optical clarity, and higher bioavailability [54,55].

Microemulsions are colloidal dispersions of oil and water stabilized by a surfactant, usually in conjunction with a co-surfactant. Microemulsions exhibit desirable characteristics such as spontaneous formation and therefore minimal energy consumption, small particle size, and physicochemical stability under specific conditions (e.g., specifically composition and temperature). They consist of an oily phase, water, and surfactant (often more than one surfactant and more than one solvent). Their preparation is easier than that of nanoemulsions (often by simple mixing), but they require higher amounts of surfactants, approximately 10–30% *w*/*w* [54].

In Pickering emulsions, solid particles are strongly anchored at the oil–water interface due to partial wetting of their surfaces by water and oil. They maintain the essential features of traditional emulsions but are more resistant to emulsion destabilization processes such as coalescence, flocculation, and Ostwald ripening, and no surfactants are necessary, allowing them to be employed in a variety of applications [50].

Producing an emulsion may require significant energy input due to the large surface area of its numerous droplets [55]. Methods for preparing emulsions can be generally divided into two main categories based on their energy requirements: “high-energy” and “low-energy” approaches (Table 2):

a. High-energy methods rely on applying high shear forces to disperse the discontinuous phase into microscopic droplets. These are as follows:
  i High- or Ultra-High-Pressure Homogenization (HPH or UHPH) creates small droplets by forcing a coarse emulsion through a high-pressure valve [56], generating fine particles of the dispersion phase [54].
  ii Microfluidization is an emulsification technique where a pre-formed emulsion is subjected to high pressure and forced through precisely designed microchannels. This process divides the emulsion into multiple microstreams that collide with each other, generating intense shear and impact forces [57,58].
  iii Ultrasonication, in which high-frequency sound waves generate microscopic bubbles in the liquid, which rapidly collapse, creating intense local turbulence and shear forces. This turbulence effectively breaks down droplets, resulting in smaller and more uniform particle sizes [59,60].
b. Low-energy methods require minimal energy because they utilize the system’s inherent chemical energy (or chemical potential of components). These are as follows:
  i The Phase Inversion Temperature (PIT) method takes advantage of the temperature-dependent behavior of certain emulsifiers, which can shift between hydrophilic and lipophilic properties. By carefully controlling the temperature, this technique induces a phase inversion, resulting in the formation of nanoemulsions with highly stable and uniformly sized droplets [54,61].
  ii The Phase Inversion Composition (PIC) method is a process that involves altering the composition of a system by introducing substances such as electrolytes or alcohols. These additives modify the properties of the emulsifier, thereby triggering a phase inversion that results in the formation of nanoemulsions [62].
  iii Spontaneous emulsification, also known as the Ouzo effect, is a process in which rapid changes in interfacial tension result in the spontaneous formation of small droplets as a solvent phase mixes with an immiscible phase [61,63,64].
  iv Emulsion Inversion Point (EIP) technique involves the creation of a water-in-oil (W/O) emulsion with a high oil-to-water ratio. As water is gradually added, a critical point is reached where the water content exceeds the oil content, causing a phase inversion from W/O to oil-in-water (O/W) emulsion [54,61].
  v Membrane emulsification is an energy-efficient technique in which the dispersed phase is forced through the pores of a specialized membrane, thereby generating droplets on its surface. These droplets are then detached and carried away by the flow of the continuous phase or through the rotation of the membrane itself [65].

**Table 2:** Low- and high-energy methods employed to generate the different types of emulsions.

Method	Energy	Macroemulsions	Nanoemulsions	Microemulsions	Pickering Emulsions
Simple Stirring/Shaking	low	✓	✗	✗	✓ ^1^
Magnetic Stirring	low	✓	✗	✗	✓
High-Speed Homogenization (HPH)	high	✓	✓	✗	✓
High-Pressure Homogenization	high	✓	✓	✗	✓
Microfluidization	high	✗	✓	✗	✓
Ultrasonication	high	✗	✓	✗	✓
Phase Inversion Temperature (PIT)	low	✗	✓	✓ ^2^	✗
Spontaneous Emulsification	low	✗	✓	✓ ^2^	✗
Emulsion Inversion Point (EIP)	low	✗	✓	✗	✗
Membrane emulsification	low	✗	✓	✗	✓

^1^ Only for preliminary mixing before applying high-energy methods. ^2^ Microemulsions can only be formed within a specific range of concentrations of oil, water, and surfactant or surfactants [66].

The use of emulsions to protect food against foodborne pathogens necessitates long-term stability. Other characteristics, such as appearance or rheological properties, are directly or indirectly related to the nanoemulsion’s stability and the final product’s organoleptic properties. Finally, the degree of encapsulation of the antibacterial ingredients (nisin) and the rate of their release into the food should be examined. The particle size in a nanoemulsion impacts its optical, rheological, stability, and release properties. The particle size distribution (PSD) of an emulsion can be controlled by adjusting the preparation conditions and system composition. In high-energy processes, droplet size is influenced by energy intensity and duration, emulsifier type and concentration, interfacial tension, and viscosity of the dispersed and continuous phases [48].

Microemulsions are thermodynamically stable under specific conditions, while coarse emulsions exhibit poor physicochemical stability. Nanoemulsions are thermodynamically unstable but kinematically stable systems. Because of their interfacial tension (1–10 mN m^−1^) and enormous surface area, emulsions have a large positive interfacial free energy (Δ*G*) and, to minimize it, they tend to increase their particle size [48,49,67,68].

The destabilization of emulsions can be caused by many different physicochemical processes. Creaming occurs due to density differences between the phases, causing lighter oil droplets to rise to the surface. Similarly, precipitation operates on the same principle but is typically observed in water-in-oil emulsions, where denser water droplets settle at the bottom. Both creaming and precipitation can be mitigated by increasing the viscosity of the continuous phase, which hinders droplet movement.

Particle collisions within an emulsion are inevitable due to relative motion. These collisions can lead to flocculation, when emulsion droplets aggregate into clusters without rupturing the interfacial membrane, and to coalescence when droplets come into contact, the interfacial membrane ruptures, causing the droplets to merge and their contents to fuse.

While creaming and flocculation do not directly increase droplet size, they often precede coalescence, which is widely regarded as the most critical mechanism of emulsion destabilization. Coalescence can be prevented by carefully selecting stabilizers such as Texture Modifiers or Weighting Agents that restrict Brownian motion [54].

Ostwald ripening follows a distinct pathway. It occurs when molecules from smaller dispersed droplets dissolve into the continuous phase and then diffuse toward larger droplets and are absorbed by them. This process causes larger droplets to grow at the expense of smaller ones. The driving force behind Ostwald ripening is the difference in Laplace pressure, which is higher in smaller droplets due to their greater curvature. This pressure gradient promotes mass transfer from smaller to larger droplets, ultimately leading to emulsion instability [69].

Emulsion characteristics may be assessed by several physicochemical methods. The most investigated parameters concerning emulsion stability are particle size, size distribution, ζ-potential, and viscosity. Particle size is connected to the emulsion’s susceptibility to gravitational separation. Emulsions containing large particles are prone to gravitational separation, while those with a particle diameter of less than 50 nm are very stable [70]. The most common method to determine particle size is Dynamic Light Scattering (DLS). A monochromatic beam of light is focused on the sample. The intensity of the scattered beam versus time varies with particle size because larger droplets move more slowly than smaller droplets due to Brownian motion. From the variation in intensity of the scattered beam, a correlation coefficient is calculated, from which the hydrodynamic diameter of the particles using Stokes–Einstein theory is determined [71].

Small-angle X-ray Scattering (SAXS) is a powerful non-destructive method for the determination of an object’s size, size distribution, shape, and surface structure. It is based on the scattering pattern of X-rays at small angles (0.1° to 10°) [72] to investigate structural features on a scale of 1–100 nm [73]. SAXS is ideal for studying solid, liquid, or gel samples. Macromolecules that can be studied range in size from tiny peptides or snippets of nucleic acids to complexes with sizes in the range of Giga Daltons or even whole viruses [72].

Multi-Angle Laser Light Scattering (MALLS) is a technique for measuring larger particles. According to Mie’s theory, the scattered light is anisotropically dispersed and becomes increasingly concentrated in the forward direction as particle size increases. The scattering pattern (scattered light intensity vs. scattering angle) depends on particle size, structure, and wavelength of the radiation when detectors are placed at varying angles in relation to the original direction of incoming radiation. The particle size distribution is estimated using software that is based on the Mie mathematical model and can compare the observed scattering pattern with the theoretically anticipated one, for a particular particle size and distribution [74]. It is usually combined with a particle separation technique, such as size-exclusion chromatography.

Electron Microscopy can be employed for direct observation and evaluation of nanoscale materials. In addition, the particles’ size can be measured, and detailed information about their morphology and a clear picture of aggregation and coalescence phenomena can be obtained. The techniques most often employed are Transmission Electron Microscopy (TEM), and, more often, Cryo-TEM, Scanning Electron Microscopy (SEM), or Atomic Force Microscopy (AFM). The advantage of these techniques is undoubtedly their higher resolution, but they require access to expensive equipment and laborious sample preparation [75]. Optical Microscopy is also a rapid non-invasive technique for a first assessment of the size and distribution of particles in an emulsion. Ιt is usually combined with more targeted strategies.

Fourier-Transform Infrared Spectroscopy (FT-IR) is a spectroscopic technique that can be used for the physicochemical characterization of emulsions [76]. FT-IR can provide molecular insights into the composition and stability of emulsions, as well as the molecular interactions that take place among the different constituents. Emulsification processes, oxidation, and phase separation can be monitored though the analysis of spectral shifts. It is a rapid, non-destructive method that is valuable for quality control and formulation studies in the different food emulsions.

Measuring the absorbed UV-Vis radiation from an emulsion is indicative of the particle size. The smaller the particles, the less UV-Vis radiation is absorbed [71]. The study of Sanhcez-Ortega et al. confirmed that particle size significantly affects the transparency of emulsions. Emulsions with particles smaller than 100 nm in diameter were more transparent, while those with larger particles (>100 nm) were more opaque and less stable [77]. Also, changes in turbidity or transparency due to coalescence and aggregation phenomena indicate that the emulsion is destabilized.

Electron Paramagnetic Resonance (EPR) is another powerful technique for the physicochemical characterization of emulsions [78]. It can shed light on molecular dynamics, polarity, and microenvironmental changes. EPR detects unpaired electrons in spin probes or radicals and can assess emulsion stability and surfactant behavior. EPR has high sensitivity, making it a valuable tool for studying emulsion stability in food formulations.

DLS devices may also determine the polydispersity index (PDI), which represents the particle size distribution. The wide particle size distribution facilitates Ostwald ripening, which results in increasing particle diameter over time. But in addition to size heterogeneity, polydispersity can result in flocculation or coalescence. According to international standards organizations (ISOs), values > 0.7 indicate a broad distribution of particles (polydisperse), whereas values < 0.3 are more prevalent in monodisperse samples (ISO 22412:2017) [79,80].

Another critical parameter reflecting emulsion stability is the *ζ*-potential (zeta potential). It is the measure of the electric potential at the hydrodynamic plane of shear, which includes the nanoparticle and a layer of counterions firmly attached to its surface. The ζ-potential determines the electrostatic repulsion between particles, which directly affects the emulsion’s resistance to aggregation and coalescence. The ζ-potential can be either positive or negative, depending on the surface charges of the particles. Higher absolute values of the ζ-potential indicate stronger electrostatic repulsion between particles. As a general rule, systems with a ζ-potential greater than +30 mV or less than −30 mV are considered stable, while those with values within the ±30 mV range are more prone to instability [81].

The viscosity of the emulsion is another important parameter. Increased viscosity contributes to the final product’s stability by inhibiting particle mobility and thus delaying gravitational separation, aggregation, or coalescence. ’Stabilizers’, or compounds that function as ’thickening agents’, are frequently added to increase the viscosity of the continuous phase. Their use reduces the rate of droplet collision and precipitation [82,83,84]. Rheological measurements in the characterization of emulsions are also required to predict their behavior during the manufacturing process (flow through tubes, ease of blending), packaging, or final use, where the consumer’s choice is intimately related to the texture of the product [85,86].

Furthermore, the emulsion’s optical characteristics vary with particle size. The opacity of a nanoemulsion is determined by the scattering of light from oil droplets. The amount of light scattering from an emulsion is determined by the size of the droplets in relation to the wavelength of the light, with the most intense scattering happening when the droplet diameter equals the wavelength. Droplet sizes in nanoemulsions range from 20 to 200 nm, allowing for a wide range of appearances, from clear (smaller in size droplets) to opaque (larger droplets) [87]. The total number, size, properties, and placement of the droplets in a nanoemulsion determine its final appearance, as do any other components that distribute or absorb light. [81,88]. The afore mentioned methods are summarized in Table 3. 

**Table 3:** Methods of physicochemical characterization of emulsions as carriers of nisin.

Method	Purpose	References
DLS	Particle size and polydispersity index	[77,89,90,91,92,93,94,95,96,97,98,99]
Small Angle X-ray scattering (SAXS)	Micelles’ structure	[92]
ζ potential	Electrophoretic mobility	[77,89,99,100,101]
SEM	Particle morphology	[96]
TEM	Particle morphology	[89,93,98,101,102]
Optical Microscopy	Emulsion morphology	[98,102]
FT-IR	Chemical structure and intermolecular interactions	[93,96,98]
UV-VIS	Turbidity (coalescence and aggregation phenomena) or transparency	[77,93,95,96]
Electron Paramagnetic Resonance (EPR)	Interfacial properties	[77,90,91,92,93,95,96]
High-Performance Size-Exclusion Chromatography-(HPSEC)-Multi-angle Laser Light Scattering (MALLS)	Weight-average, molecular masses-root mean, square radius (R_Z_)	[99,101,103]
Electrical conductivity measurements	Information about the continuous phase in w/o microemulsions	[42,91,92]
Rheological Analysis	Viscosity	[45,90,102]

## 5. Encapsulation Systems of Nisin Based on Emulsion

### 5.1. Coarse Emulsions

The Maillard reaction has been used to change protein physical characteristics such as solubility, emulsification capacity, and antibacterial activity by glycosylating them with reducing sugars. Nisin is a cationic amphiphilic peptide. Glycosylated nisin has a lower positive charge and hydrophobicity and interacts less with food components, potentially increasing antibacterial action in food matrices. However, because nisin’s action against Gram-positive bacteria is derived from positive charges localized in lysine residues, which are also the primary places for the Maillard process, glycosylation is expected to impede nisin’s antimicrobial activity [104]. Samples of glycosylated nisin were produced with a disaccharide (lactose), an oligosaccharide (maltodextrin), and a polysaccharide (dextran) to investigate the physicochemical properties of nisin after glycosylation. This study aimed to determine the antibacterial activity of glycosylated nisin emulsions, compared to non-glycosylated nisin, and the effectiveness of a mixture of nisin and thyme oil emulsions against the Gram-negative bacteria *Micrococcus luteus* ATCC 10240. ATCC 43895, 43889, 43894, and K3995 strains, *Escherichia coli* O157:H7, and two Gram-positive bacteria, *L. monocytogenes* Scott A and *Bacillus subtilis*. The degree of glycosylation was reduced as the sugar chain length grew, while residual nisin efficacy against *M. luteus* ATCC 10240 increased. Testing against Gram-positive *L. monocytogenes* Scott A and *B. subtilis* revealed that the MIC and MBC for the same nisin units were similar. However, it failed to prove effective against *E. coli* O157:H7. In TSB, the combination of thyme oil and nisin, whether glycosylated, free, or emulsified, had a clear additive effect against Gram-positive bacteria. However, when thyme essential oil emulsions incorporating glycosylated nisin were mixed with 2% fat milk, the antilisteric action was lower than when natural nisin and free thyme oil were combined in equal amounts. This suggests the glycosylation of nisin is an inefficient way to enhance its antilisteric action.

Biofilms are communities of bacteria, as well as other microorganisms, in which bacteria are discovered encased in a matrix of extracellular polymeric substances generated by themselves [105]. *Listeria monocytogenes* can form biofilms within food-processing environments, potentially leading to contamination and foodborne illness. Hossain et al. created emulsions using single or combinations of current antibacterial GRAS components (nisin, thymol, and eugenol) at various concentrations (lower or higher than the MIC), Tween 80 as an emulsifier, and DMSO as a solvent. They evaluated each emulsion’s ability to inhibit biofilm formation by *L. monocytogenes* on rubber gloves, lettuce leaves, and MBEC™ biofilm device (simulation of food-processing surface). The addition of EO components (thymol or eugenol at 0.5, 1, and 2 MIC) to nisin (at 250 or 500 IU/mL) significantly reduced the formation of *L. monocytogenes* biofilms, demonstrating a synergistic effect, although the mechanism of this was not elucidated [106].

### 5.2. Nanoemulsions

D-limonene (4-isopropenyl-1-methylcyclohexene) is present in many citrus-derived essential oils and is generally considered safe (GRAS) for use as a flavoring and food preservative. Its antibacterial action is proven against food-related microorganisms, including *St. aureus*, *L. monocytogenes*, and *Salmonella enterica* [107]. Due to its hydrophobic nature, D-limonene must be used in significant quantities to obtain effective antibacterial activity in food. Furthermore, D-limonene is prone to oxidative decomposition, resulting in a loss of action [108].

Zhang et al. prepared a series of nanoemulsions encapsulating D-limonene alone or in combination with nisin, allowing for the use of a smaller amount of D-limonene in order to not affect the organoleptic characteristics of the food while also addressing the problems of its solubility and oxidation [95]. The catastrophic phase inversion method was utilized for the preparation of nanoemulsions. The aqueous phase consisted of water, propylene glycol, and nisin, while the oily phase was a mixture of Tween 80 and D-limonene. The antimicrobial activity of the nanomaterials was tested against the Gram-positive bacteria *St. aureus* ATCC6538 and *B. subtilis* ATCC 6633, the Gram-negative bacterium *E. coli* ATCC 8739, and the yeast *Saccharomyces cerevisiae* ATCC 9763. When applied individually, D-limonene demonstrated strong antimicrobial activity against all four microorganisms, whereas nisin was active against Gram-positive bacteria. However, when used in combination, synergistic activity was observed, as evidenced by the FIC index. The MICs of nano-encapsulated D-limonene, whether alone or combined with nisin, were all lower than those of unencapsulated D-limonene against all microorganisms. The MIC of nisin was also significantly lower for all the target microorganisms. Scanning Electron Microscopy (SEM) confirmed that nisin-D limonene nanoemulsion targeted the cell membrane.

Maté et al. studied the antibacterial efficacy of combining nisin and D-limonene against *L. monocytogenes* in tryptic soy broth (TSB) (at pH 7.3 and 5.5), vegetable cream (pH 5.6), and chicken broth (pH 6.8) [94]. The antimicrobials were administered to the medium singly or in combination, directly or as nanoemulsions, and their effects on growth or viability were monitored. The catastrophic phase inversion method, introduced by Zhang et al., was used for producing D-limonene emulsions or combinations of D-limonene and nisin. After six months of storage, the nanoemulsions remained stable, with no significant change in particle size. According to the optical density growth curve data, D-limonene in a nanoemulsion improved the antibacterial action when administered directly to the growing medium. A comparable result was achieved by mixing both antimicrobials into a nanoemulsion, which stopped growth for more than 100 h. The results also indicated that D-limonene was less effective at an acidic pH. After 28 days, the viability of microorganisms was evaluated, and no growth was detected in any medium to which the combination of nisin and d-limonene was administered, either as a solution or as a nanoemulsion.

In a recent study, the impact of different vegetable oils (olive, corn, and sesame) on the stability and properties of oil-in-water (o/w) nanoemulsions in the presence and absence of nisin and limonene was investigated [109]. Nanoemulsions were prepared using various oil concentrations (2%, 5%, 10%, and 15% *v*/*v*), with nisin incorporated as a Nisaplin aqueous solution. The formulations were homogenized at high speed, and their physicochemical properties were assessed.

The results indicated that oil type significantly influenced emulsion stability. Sesame-oil-based nanoemulsions exhibited stable droplet sizes and lower PDI values, while olive-oil-based emulsions performed better at lower concentrations. Nisin reduced droplet size, potentially due to its amphiphilic nature, whereas the presence of limonene increased droplet size. The combined use of nisin and limonene resulted in intermediate droplet sizes. Nanoemulsions with 2% oil remained stable for 15 days, while higher concentrations led to phase separation. Ultimately, sesame-oil-based nanoemulsions demonstrated favorable physicochemical properties, but practical stability assessments favored olive-oil-based formulations.

The study by Pagnossa et al. demonstrates the significance of how natural compounds are delivered as antimicrobial agents in preventing sublethal tolerance to pathogenic germs. In their study, the synergistic activity of nisin and three essential oils, cinnamaldehyde (CIN), citral (CIT), and linalool (LIN), against *Bacillus cereus* UC-4044 was assessed either as a mixture or as nanoemulsions. The nanoemulsions contained CIN, CIT, or LIN with or without added nisin, and were prepared via ultrasonication with Tween 80 as a surfactant. The particle size distribution (PDI), mean particle diameter (nm), and zeta potential (z) were all measured. After 30 and 90 days of storage at 25 °C, the samples were examined and found to be stable. TEM microscopy demonstrated morphological alterations in the structure of B. cereus when treated with cinnamaldehyde (CIN) and citral (CIT) nanoemulsions in conjunction with nisin. Furthermore, sensitivity tests and UHPLC-QTOF-MS analyses showed that while, in solution, nisin can react with citral, reducing its bactericidal efficacy, in the nanoemulsion, its activity is, instead, enhanced [110].

Medium- and long-chain fatty acids are mostly sourced from vegetable oils and animal fats. They are frequently used as preservatives to avert food degradation due to their excellent antibacterial characteristics and safety [111]. However, because fatty acids are not soluble in water, their efficiency may be diminished. Guo et al., in their study, focused on the synergistic antimicrobial activity of a nanoemulsion in which hydrophilic nisin and lipophilic octanoic acid (NOA) were encapsulated against *E. coli* and *St. aureus* in vitro. Its physicochemical properties were also investigated and were compared to those of free nisin and octanoic acid, respectively [93]. The nanoemulsions were prepared via ultrasonication and a combination of Tween 80 and Span 85 with HLB 13.5 was used as an emulsifier. According to the DLS method, the average particle size of the NOA nanoemulsion was 52.21 ± 0.43 nm with a polydispersity index (PDI) of 0.25 ± 0.01, indicating uniform dispersion. TEM images also showed that the droplets of NOA nanoemulsion were spherical. The MIC and MBC of nisin against *E. coli* in the NOA nanoemulsion were reduced twofold and fourfold, (250 μg/mL και 500 μg/mL), respectively, when compared to free nisin. For *St. aureus*, the MIC and MBC of nisin in the NOA nanoemulsion were 1 μg/mL and 4 μg/mL, respectively, four-fold and two-fold lower than free nisin. The above led to the conclusion that the required amount of nisin could be reduced by octanoic acid in a nanoemulsion and that the presence of the acid may enhance nisin’s antimicrobial activity against Gram-negative bacteria like *E. coli*.

Sadiq et al. combined nisin Z and monolaurin to form food-grade nanoemulsions, via ultrasonication, with synergistic antimicrobial effects against *St. aureus*. Monolaurin is GRAS and is often used as an emulsifier and antimicrobial agent. The mean diameter of nisin-loaded monolaurin-based nanoparticles (MNPs) was 59.19 ± 8.66 nm compared to 54.7 ± 5.6 nm for empty MNPs. The zeta potential values were −45.1 ± 2.6 mV and −40.5 ± 2.12 mV, respectively. Reversed-phase HPLC was the selected method for determining the encapsulation efficiency of nisin. The samples are centrifuged, and the supernatant is passed into a reversed-phase HPLC column. The nisin content is calculated from a standard curve constructed following the same procedure using aqueous nisin solutions of known content. The encapsulation efficiency of nisin was 78.8 ± 2.5%, and according to the FTIR study, it was solely due to electrostatic attraction. Furthermore, nisin-loaded MNPs inhibited the growth of *St. aureus* ATCC-25923 in a synergistic manner for 164 h [100].

### 5.3. Microemulsions

In their study, Chatzidaki et al. prepared microemulsions using DMG as an emulsifier and mixtures of refined olive oil (ROO) or sunflower oil (SO) and ethanol in varying quantities as the oil phase [92]. The emulsifier–oil phase ratio was constant at 40: 60. It was observed that increasing ethanol mass fraction in the oil phase (Wethanol) decreased the size of the reverse micelles, which was attributed to the incorporation of ethanol at the oil–aqueous phase interface, among the emulsifier molecules, but simultaneously increasing its fluidity according to both EPR and SAXS measurements. It was also found that the ROO microemulsion shell was more structured, probably due to the presence of monosaturated oleic acid. Increasing the water content from 0 to 9% *w*/*w* also increased the size of the particles. The antimicrobial activity of microemulsions was evaluated with a Well Diffusion Assay against *L. lactis* MG1363 culture. Higher-ethanol-content microemulsions resulted in larger inhibition zones (*W*_ethanol_ = 0.50), whereas the nature of the oil used for the preparation of microemulsions did not affect it. This was attributed to the flexibility of the interface in the presence of ethanol, which made the diffusion of nisin easier.

In another study, the researchers sought to optimize the above-mentioned microemulsions to achieve maximum water and nisin content by adding the nisin solution dropwise until a transparent w/o microemulsion was achieved: ROO and ethanol-based systems showed the capacity to absorb slightly higher quantities of water [91]. This observation was attributed to the greater stability of ROO micelles. It was also discovered that raising the temperature from 25 to 37 °C had no discernible effects on the systems’ structural behavior. For ROO, the micelles’ size increased in the presence of nisin, whereas for SO, the difference was minor, and the total size might be regarded as being unaltered when nisin was present. Both the empty and nisin-loaded micelles of both oils displayed identical EPR spectra, indicating that the nisin was primarily positioned in their core. The antibacterial efficacy of microemulsions was tested against *St. aureus* and *L. monocytogenes* on lettuce leaves and minced beef, respectively. Ethanol gave both empty (no nisin) and loaded (nisin) systems antibacterial effects on lettuce leaves. Furthermore, the combined use of nisin and ethanol displayed a synergistic effect when applied to lettuce leaves, but the impact was less prominent in minced meat, most likely due to the interaction of ethanol with the meat component.

In a later study, the researchers replaced part of the oily phase with essential oils of oregano (OEO), thyme (TEO), rosemary (REO), and dittany (DEO). Mixtures of ROO and EO (at 2:1 *w*/*w* ratio) were used as a continuous oil phase, DMG was used as a surfactant, and water or a nisin solution were used as a dispersed aqueous phase to create w/o microemulsions [90]. Either propylene glycol (PG) or ethanol were implemented in the systems to increase the stability and dispersion of the aqueous phase. The microemulsions were prepared using ethanol in smaller particles. The presence of essential oils reduced the viscosity and enabled the researchers to reduce the amount of emulsifier from 38 to 22 and 19%. As a result, the micelle membrane became more elastic, facilitating the diffusion of nisin. Nisin microemulsions were evaluated for antibacterial activity against *L. lactis* MG1363, as well as three Gram-positive foodborne pathogens: *St. aureus* FMCC B-95, *L. monocytogenes* FMCC B-128, and *B. cereus* LMG 6923T. Most of the tested bacterial strains responded best to DEO-based microemulsions, while ethanol-containing systems demonstrated a stronger antibacterial impact than PG-based systems. Finally, microemulsions with lower percentages of DMG (22 and 19%) had stronger antibacterial activity than those with 38% DMG, despite presenting a smaller percentage of aqueous phase and thus nisin molecules.

### 5.4. Pickering Emulsions

Bi and colleagues prepared o/w nisin emulsion stabilized with octenyl-electrolyte Phyto glycogen (PG-OS) to extend the efficacy of bacteriocin against food bacteria, particularly *L. monocytogenes* [89]. PG-OS is an amphiphilic, negatively charged, dendrimer-like polysaccharide that is partly digested. It can produce emulsions with great physical and oxidative durability [112]. PG-OS and WCS-OS (waxy-corn starch octenyl succinate) and Tween 20 as emulsifiers and high-pressure homogenization techniques were used. The dynamic light-scattering technique revealed that all PG-OS nanoparticles in the emulsion adsorbed on the oil droplet surface, and zeta potential measurements showed that positively charged nisin molecules were effectively adsorbed on the surface of PG-O molecules at the interface. Compared to WCS-OS and Tween 20, the PG-OS stabilized emulsion was more effective in prolonging nisin’s activity against the pathogen (up to 40 days). Furthermore, the structure of PG nanoparticles may be manipulated via biological, chemical, and enzymatic methods to generate a new class of particles with uses other than food.

Sarkar et al. investigated the interactions between adsorbed nisin and PG-OS by comparing its mode of adsorption in emulsion and non-emulsion systems [101]. The study found that the quantity of nisin in the emulsion system is higher and is related to the substitution degree of PG-OS. It was also consistent with a Langmuir monolayer model. The authors evaluated the adsorption of nisin in emulsion using equilibrium dialysis. Non-adsorbed nisin molecules were sampled using the fast micro-equilibrium dialyzer (FMED) unit (Harvard Apparatus, Holliston, MA, USA). The concentration of adsorbed nisin was 157 and 22 μg/mL in the emulsion and non-emulsion systems, respectively, when the same dosages of PG-OS (5.0 mg/mL) and nisin (200 μg/mL) were used. It was assumed that the PG-OS particles were distributed equally on the surface of the particle. As a result, it was concluded that, in addition to the electrostatic attraction between the nisin and octenyl succinate groups, there are hydrophobic interactions and adsorption of nisin on the exposed surface of the oily phase.

In another study, Sarkar et al. came to a similar conclusion after employing encapsulation to protect nisin from proteolytic degradation in an o/w emulsion stabilized with starch-OS, testing its efficiency against *L. monocytogenes* in cantaloupe juice, and investigating its mode of action [99]. Starch-OS was chosen instead of PG-OS because it costs less and is commercially available. In the food industry, starch-OS is frequently used as an emulsifier, encapsulating agent, and fat replacement [113]. O/w emulsions were produced using the high-pressure homogenization technique. The added nisin was adsorbed on the surface of the micelles as determined by ζ-potential measurements. It was also found that encapsulated nisin was protected from the proteases of cantaloupe juice and stayed effective for almost 6 days in cantaloupe juice. On the other hand, when free nisin was added to the juice nisin, it was deactivated after 2 days of storage.

In another study performed by Sarkar et al., o/w emulsions stabilized with starch octenyl succinate (starch-OS) were used as a delivery system for nisin and thymol in cantaloupe juice [114]. Antimicrobial tests were conducted against two model pathogens: *L. monocytogenes* V7 (a Gram-positive bacterium) and *S. enterica* serovar Typhimurium (a Gram-negative bacterium). The results demonstrated that the emulsions significantly enhanced the retention of nisin and thymol, leading to prolonged antibacterial efficacy compared to non-emulsion, aqueous formulations. The study also found that the combination of nisin and thymol in emulsions exhibited synergistic effects, particularly against *L. monocytogenes*. For *Salmonella* Typhimurium, the emulsion system enhanced the antibacterial efficacy of thymol, and the addition of nisin further improved the inhibitory effect. Thymol, which is effective against Gram-negative bacteria, likely disrupted the outer membrane of Salmonella, making it more susceptible to nisin.

### 5.5. Double Emulsions

Luo et al. developed water-in-oil-in-water (W/O/W) emulsions with a water core containing nisin, Momordica charantia extract (MCE), and nisin-producing *Lactobacillus plantarum* as functional additives, maize oil as an intermediate wall, and rock bean protein (RP)/Arabic gum (GA) for the outer shell. Span 80 was used as an emulsifier. The microcapsules were then mixed into flour to create a form of enriched noodles. The properties of the microcapsules and the effect of integrating them into the noodles were examined [115]. When the oil-to-water ratio was 5:1, the encapsulation rate of the aqueous phase in the precursor w/o emulsion reached 28 ± 0.34%. The lowest microcapsule size was found when the RP/GA ratio was 1:1. The addition of nisin via microcapsules was shown to prolong the shelf life of fresh pasta, while 3% *w*/*w* microcapsules were shown to inhibit the formation of bacteria and mold.

### 5.6. Use of Nanoemulsions for the Preparation of Other Delivery Systems

#### 5.6.1. Organogels

Gels are semi-solid structures consisting of a gelling agent (organogelator), which forms a three-dimensional network, and a liquid phase entrapped within their structure. Gels are classified as either hydrogels or organogels. An organogel is made up of an organic liquid entrapped inside a three-dimensional gel network that is thermo-reversible, anhydrous, and viscous elastic. The gel network is formed by the self-assembly of a low concentration of organogelator molecules with low molecular weights capable of gelling organic liquids [116,117].

The term ’emulsion gel’ is a convenient term for soft-solid substances. Two structural arrangements describe the spatial distribution of particles. In the emulsion-filled protein gel, the droplets are embedded within a protein matrix, while in the protein-stabilized emulsion gel, the droplets themselves form the network. The emulsion-filled protein gel is a protein gel matrix in which emulsion droplets are embedded, whereas the protein-stabilized emulsion gel is a sort of particulate gel in which the network is made up of partly aggregated emulsion droplets and the protein molecules help to stabilize this network [118].

Bei et al. combined the advantages of organogel and nanoemulsion and prepared an organogel-based nanoemulsion in which they encapsulated D-limonene and nisin [119]. Initially, the organogel was made of stearic acid, sucrose stearate, peanut oil, nisin, and D-limonene and was converted into a nanoemulsion by the high-pressure homogenization technique, using Tween 80 as an emulsifier. The organogel-based nanoemulsion increased the solubility and stability of hydrophobic compounds. Although the surfactant-to-oil ratio (SOR) was kept low (1:8), the preparations had a narrow particle size distribution and high storage stability. The combination of D-limonene and nisin was effective against Gram-positive bacteria *St. aureus* ATCC6538 and *B. subtilis* ATCC6633, and Gram-negative bacteria *E. coli* ATCC8739. SEM microscopy of the bacterial cells demonstrated that the organogel-based nanoemulsion of D-limonene with nisin disrupted the structure of the cell membrane, resulting in the death of microorganisms [119].

#### 5.6.2. Nano/Microcapsules

Ji et al. produced hydrophobic poly lactic acid (PLA) particles loaded with nisin [120]. Their size and shape were controlled and the water-in-oil-in-water emulsion process was used. Initially, EtOAc (oil phase), poloxamer Pluronic F68 (PF68), and glycerol were used to adjust its viscosity, and nisin was dispersed into water (continuous phase). PLA solution in water-saturated EtOAc was added, and the mixture was stirred overnight. Depending on temperature during the emulsification process, plain PLA can form hollow microspheres (1–5 μm) at 60 °C, whereas solid nanospheres (30–100 nm) can be formed at room temperature. Furthermore, the researchers showed that alternating temperatures between 60 °C and 25 °C can cause a transition between the two structures and, thus, and that by adjusting the timing of phase transition, they could control the particle size, shape, and amount of encapsulated nisin. Confocal laser scanning microscopy (CLSM) revealed that the hydrophilic nisin can be present on the surface or inside the polymer particles. Zeta potential of plain PLA particles (−18 mV) increased to +5 mV, confirming the presence of nisin on the particle surface. The encapsulation efficiency was assessed by RF-HPLC, and it was found improved as the nisin concentration in the aqueous solution was increased.

In their study, Sangcharoen et al. utilized the combination of nisin, ascorbic acid, and EDTA, followed by encapsulation via double emulsion and freeze-drying techniques [121]. The encapsulation significantly enhanced nisin’s antimicrobial activity, particularly against Gram-negative bacteria, achieving reductions of 24.74–100%. The microcapsules (MCs) formed had high encapsulation efficiency (44.40%) and a controlled release profile, allowing for prolonged antimicrobial activity. Encapsulated nisin completely inhibited *Salmonella* Enteritidis in culture broth after 4 days and reduced its presence in minced fish by 1.5 log CFU/g after 8 days of storage at 4 °C.

The objective of the study by Calderón-Oliver et al. was to determine the conditions for encapsulating nisin and avocado antioxidant extract via complex coacervation [122]. The study assessed two matrix wall systems (collagen-alginate and collagen-pectin), two drying methods (freeze and spray drying), and two core dispersion systems (W/O emulsion or suspension). The emulsions were prepared by forming a double emulsion (W/O/W), where the active ingredient was dispersed in a primary water-in-oil (W/O) phase and then surrounded by a solution of collagen and polysaccharides (pectin or sodium alginate). Adjusting the pH to 3 enhanced the electrostatic interactions between collagen and polysaccharides, leading to the formation of stable microcapsules. The primary findings of this study indicated that the microcapsules obtained through the freeze-drying process exhibited an amorphous structure, characterized by sharp edges, while the combination of spray-drying with emulsification produced concave microcapsules with smoother surfaces and enhanced encapsulation yield (69.28–73.69%). In contrast, the particles with the non-emulsified core, combined with freeze-drying, yielded the highest loading of active ingredients. However, in this case, the proximity of the ingredients to the capsule’s surface can result in their rapid degradation or release into the environment.

Pinilla et al. prepared polymeric nanocapsules with a lipid core using biodegradable polymers such as poly(e-caprolactone) (PCL), Eudragit RS-100^®^ (EUD), and poly(butylene adipate-co-terephthalate) (PBAT) [123]. The encapsulation efficiency of nisin in polymeric nanocapsules was extremely high, with values above 96%, suggesting that nisin remains stable and active within the nanocapsules during the preparation process. The EUD and PCL nanocapsules had a smaller size (145–303 nm) and narrower size distribution (PDI ~0.3), while the PBAT nanocapsules had a larger size (556 nm) and wider distribution (PDI ~0.51). All nisin-containing nanocapsules demonstrated antimicrobial activity against *L. monocytogenes*, with EUD-containing nanocapsules showing the largest zone of inhibition (13.5 mm) in agar diffusion assays, in contrast to 9.5 mm of free nisin. In liquid medium assays, PCL showed the fastest release of nisin (400 AU/mL), whereas the nanocapsules with EUD and PBAT had the slowest release (200 AU/mL and 100 AU/mL, respectively). Thermogravimetric analysis (TGA) showed that nisin decreased the decomposition temperature of the nanocapsules with PCL and EUD, while it increased the thermal stability of the nanocapsules with PBAT.

Nisin-loaded chitosan/sodium alginate microspheres were prepared using the water-in-oil emulsion cross-linking process by Tang et al. and tested for antibacterial activity against *St. aureus* [124]. Microspheres reduced the minimum inhibitory concentration (MIC) against *St. aureus* by 50% compared to free nisin, with an encapsulation effectiveness of 87.60%. The nisin release was higher at 37 °C and alkaline pH.

#### 5.6.3. Film/Coating-Based Delivery Systems

An edible coating (EC) is a thin layer of edible material applied as a coating to a food product, whereas an edible film (EF) is a thin layer of edible material constructed in advance that, once made, may be deposited on or between food components. The primary distinction between these food systems is that EC is applied to the food in liquid form, typically by immersing the product in a solution containing its structural matrix (carbohydrate, protein, lipid, or multi-component mixture), whereas EF is molded as solid sheets and then applied as a wrapping to the food product [125].

Hashemi et al. combined thymol and nisin in an edible nano-gelatin coating and assessed its antibacterial effects on the chemical quality of rainbow trout samples maintained at 4 °C for up to 16 days [126]. The coating was made of gelatin (5% *w*/*w*), glycerol (0.75 mL/g) as a plasticizer, nisin, and thymol to give final concentrations of 250 and 500 ppm, respectively. The emulsion initially obtained from a digital mixer was exposed to ultrasound, which led to nanogel formation. The measurement of pH and total volatile basic nitrogen (TVB-N) showed that the emulsions containing nisin and thymol 05 were the most effective in antibacterial protection and probably worked synergistically. Peroxide value (PV), thiobarbituric acid reactive substances (TBARS), and free fatty acids (FFA) analysis demonstrated that adding thymol to nanogelatin slowed lipid oxidation.

Eldib et al. prepared edible films of chitosan and 1% nano-silicon dioxide while incorporating 1% nisin to protect blueberry (*Vaccinium myrtillus*) at ambient temperature [127]. Chitosan (CHN), chitosan-silica (CHN-Nano), and chitosan-silica-nisin (CHN-N-Nano) solutions were used to evaluate the quality of the fruit after 2, 4, 6, and 8 days of storage at room temperature. The authors found that the chitosan film coatings combined with nisin could keep the blueberries fresh at room temperature for eight days. The nano-coating films preserved the texture of the fruit and demonstrated an antimicrobial effect throughout storage. Although nanomaterial films may affect the color of the coated samples, they were successfully used to prolong the shelf life of blueberries and other consumable vegetables.

Liu et al. developed a nanoemulsion encapsulating nisin, polylysine, and star anise essential oil, which was incorporated into an edible coating (NEAC) to enhance the quality and shelf life of Yao meat [97]. The aqueous phase was prepared using polylysine, nisin, and soy protein isolate (SPI) at varying concentrations (0.5–1.5% *w*/*v*), lecithin (0.05% *w*/*w*), and water. The nanoemulsion was formed by mixing 0.4% *w*/*w* essential oil with the aqueous phase using high-speed mixing and ultrasonic emulsification. The nanoemulsion was added to a solution of Artemisia sphaerocephala Krasch—gum (ASKG) and glycerol (40% *w*/*w* of ASKG). Meat samples were immersed in different coating solutions, dried, vacuum-sealed, and stored at 4 °C for 20 days. The nanoemulsion with 1.5% SPI exhibited the highest stability, smallest particle size, and strongest antimicrobial activity. NEAC significantly improved the pH and TVB-N values of the meat while extending its shelf life from 8 to 16 days. Sensory analysis showed enhanced color, odor, and overall acceptability.

Jiang et al. incorporated a w/o/w emulsion (EN) of encapsulated nisin prepared into nanofibers of polyvinyl alcohol (PVA) and polyacrylate sodium (PAAS) via the electrospinning technique [96]. The inner phase was an aqueous solution of nisin, the oily phase was soy oil containing different amounts of Stepan^®^ 80, and the outer aqueous phase consisted of gelatin and Tween 80 as emulsifiers. The encapsulation of nisin in the emulsion was 86.66 ± 1.59%, the particle size was 320 ± 20 nm, and the polydispersity index was 0.27. According to scanning electron microscopy (SEM), the inclusion of EN increased the nanofibers’ antibacterial activity, made the network structure more compact, and improved their morphology. The greatest results were obtained using PVA/PAAS/EN-15% nanofibers. EN also doubled the tensile strength of PVA/PAAS/EN-15% nanofibers while increasing their density. They have poor water vapor permeability and light transmittance. Moreover, the release of nisin from the nanofibers reached 85.28 ± 2.38%, a rate achieved in 16 days. For the same period, the agar diffusion assay showed significant activity against *E. coli* and *St. aureus*.

Nisin—encapsulated in an o/w nanoemulsion with oleic acid as an oil phase—was incorporated into coating suspensions along with lauric acid arginate (LAE) and lactic acid (ALA) [77]. This mixture showed high efficacy against food-related microorganisms such as *Brochothrix thermosphacta*, *L. monocytogenes Scott A*, and *Micrococcus luteus ATCC 11509*, which can cause food poisoning. Nisin films were applied to food surfaces such as fresh pork, tomatoes, and nuts. The results showed that starch-based coatings, particularly those containing nisin in combination with other antimicrobials, reduced the growth of microorganisms while maintaining the aesthetic properties of the food. The mixture with 3.75 mg/mL nisin was considered to be optimal while ensuring the homogeneity and stability of the coating.

Mavalizadeh et al. studied the combination of nisin and rosemary essential oil in free form (FRR) and as a nanoemulsion (NER) as a natural preservative system for chicken fillets during refrigeration [128]. The study focused on its impact on microbial, chemical, and sensory quality over a 12-day storage period. The results showed that treatments combining nisin with rosemary essential oils (N + FRR and N + NER) were significantly more effective than individual treatments or distilled water control. Among these, the combination of nisin with rosemary nanoemulsion (N + NER) demonstrated superior antimicrobial and antioxidant properties, reflected by the lowest mesophilic bacterial count (7.19 ± 0.05 log CFU/g) and thiobarbituric acid index (0.64 ± 0.01 mg MDA/kg) after 12 days. In the second phase, a chitosan coating was applied over the N + NER treatment (OC). The chitosan further improved microbial stability, chemical integrity, and sensory properties, making OC the most effective treatment overall.

Kazemeini et al. prepared a biodegradable coating based on chitosan nano-gel/emulsion encapsulating *Bunium persicum* essential oil (BPEO) and nisin to protect trout fillet against *E. coli* O157:H7 for 12 days at refrigeration (4 °C) [129]. Chitosan, nisin, and essential oil were emulsified via ultrasonication, producing nanoparticles with a size of 242.4 nm. Combination treatments (e.g., nano-chitosan + BPEO + nisin) were more effective than single antimicrobial agents, showing synergistic effects. The maximum reduction rate was observed in the nano-gel/emulsion chitosan + nisin + BPEO treatment (1.18 log CFU/g reduction compared to uncoated samples).

Research by Yuan et al. focused on the development of an edible chitosan membrane incorporating a double emulsion of water-in-oil-in-water (W1/O/W2) to improve the protection of fresh fish [130]. The original W1/O emulsion contained an aqueous solution of nisin mixed with carbacrol and polyglyceryl-6-dioleate (PGD) as an emulsifier. This emulsion was then mixed with water and polyglyceryl-10 laurate (PGL) to form the double emulsion W1/O/W2. Finally, the double emulsion was incorporated into a chitosan solution to produce the composite membrane. Incorporation of the double emulsion showed increased membrane strength at low concentrations and helped to increase the thermal stability of the membrane at high temperatures while demonstrating significant inhibitory activity against both Gram-positive and Gram-negative bacteria. However, at high concentrations, it increased the oxygen permeability of the membrane. Application of the composite membrane to salmon fillets resulted in a significant increase in shelf life.

#### 5.6.4. Smart Packaging

The incorporation of nisin into edible films or coatings has been the subject of increasing research in food packaging applications, serving as a hurdle technology for food preservation. It has been combined with modified atmosphere packaging [131]), EDTA, [132], or films made of ethylcellulose/hydroxypropylmethylcellulose/ethylcellulose [133] but the application of nisin in its free form is costly and linked to a reduction in activity mostly due to degradation or deactivation [134].

Nanocellulose is a renewable material that is both biocompatible and biodegradable, and has a high specific surface area, flexibility, low density, and chemical inertness. The presence of hydroxyl groups on the nanocellulose’s surface allows it to be physically or chemically modified [135]. Lu et al. fabricated w/o Pickering emulsions using modified baggase nanocellulose with nisin through electrostatic interactions, preparing N-CNF complex particles. N-CNFs have both antibacterial properties and the presence of nisin increases the hydrophobicity of the material. Nisin reduced the surface tension of the emulsion to 51.61 mN/m, significantly improved the emulsification ability, and stabilized the oil–water interface. The emulsion was added into acrylate epoxy soybean (AESO—the continuous phase). Pickering emulsion was used as a casting agent to make the porous foam after it was thermally cured at 90 °C. The porosity of the N-CNF foam was greater than that of TOCNF foam (nisin-free foam), resulting in decreased thermal conductivity and improved thermal insulation. Furthermore, the antibacterial properties of nisin were preserved in the N-CNF foam, which inhibited the development of *L. monocytogenes* by 91.33%, compared to 99.21% in an identical foam created with nisin alone [98].

Pickering emulsions were also used as a template for the construction of porous foam by Chen et al. Water-soluble polyurethane (WPU) and nisin were added to 2,2,6,6-tetramethyl piperidine-1-oxyl-oxidized cellulose nanocrystals (TOCNC) to modify its surface properties. AESO was also used as the continuous phase as well as the thermal curing at 90 °C. The presence of nisin and WPU reduced the interfacial tension of TOCNC particles and foaming experiments demonstrated that the combination of WPU and nisin has a synergistic effect on increasing the stability of the gas and liquid interface of the Pickering emulsion. TCN and TCNW particles produced foams with a homogeneous pore structure, increased the specific surface area to 0.0612 μm^2^/μm^3^, and decreased heat conductivity compared to TOCNC [102].

## 6. Nisin’s Prospects in the Future

It will soon be 100 years since the discovery of nisin in 1928. Although it has dominated research more than any other bacteriocin, it continues apace, aided by advances in analytical methods and technology for the preparation and characterization of materials. In the food sector, significant steps have already been taken, but it is clear that there are several gaps in our knowledge of nisin.

Clarifying its precise mechanism of action, how it is impacted by laboratory conditions and how it interacts with food substances will lead to more focused experimental approaches. The presence of both natural and synthetic nisin variations complicates the process while also widening the research field. Nisin’s use may be expanded to other food categories for which it is not currently approved, as well as microorganisms against which it has not yet been tested. Ideally, optimal meal combinations, preparation and storage conditions, nisin concentration, and method of application should be developed. Another topic to investigate is the synergistic activity of nisin with other antibacterial compounds and/or natural preservation methods. The components of encapsulation systems, such as emulsifiers, that influence its activity deserve further investigation. There have been documented cases of bacteria developing tolerance to nisin, and understanding this mechanism is critical for the future of nisin-related research. Finally, our understanding of nisin may serve as a springboard for similar-scale study on other bacteriocins, such as pediocin, or related chemicals, such as natamycin, that are already in use in the food sector or on other bacteriocins that are awaiting permission from the appropriate authorities.

## 7. Conclusions

The antimicrobial peptide of nisin owes its popularity to its effectiveness against a wide range of microorganisms, the low likelihood of bacterial resistance development, its resilience to relatively elevated temperatures, and the fact that it is considered safe and its use in food has been approved. On the other hand, its poor efficacy against Gram-negative bacteria, its interaction with food matrix components or food additives, and its pH-dependent action have been noted. Nisin encapsulation of nisin can protect the molecule from environmental stresses and at the same time control its release rate to prolong its activity. Emulsions are simple to prepare and apply. They can be added to food directly or as a component of packaging materials, and their use can be scaled up for an industrial setting. They can be used alone or can be incorporated into other delivery systems like coating foams and others. Except for nisin, the inclusion of a wide variety of antimicrobial substances, natural or synthetic, can be used in combination with other antimicrobial protection strategies, e.g., radiation. 

## Data Availability

No new data were created or analyzed in this study. Data sharing is not applicable to this article.
